# A2 gene of Old World cutaneous *Leishmania *is a single highly conserved functional gene

**DOI:** 10.1186/1471-2334-5-18

**Published:** 2005-03-28

**Authors:** Yves JF Garin, Pascale Meneceur, Francine Pratlong, Jean-Pierre Dedet, Francis Derouin, Frédéric Lorenzo

**Affiliations:** 1 Laboratoire de Parasitologie-Mycologie, Hôpital Saint-Louis, Assistance Publique Hôpitaux de Paris, U.F.R. Lariboisière, Université Paris VII, France; 2 Laboratoire de Parasitologie et Centre National de Référence des Leishmania, C.H.U. de Montpellier, Montpellier, France

## Abstract

**Background:**

Leishmaniases are among the most proteiform parasitic infections in humans ranging from unapparent to cutaneous, mucocutaneous or visceral diseases. The various clinical issues depend on complex and still poorly understood mechanisms where both host and parasite factors are interacting. Among the candidate factors of parasite virulence are the A2 genes, a family of multiple genes that are developmentally expressed in species of the *Leishmania donovani *group responsible for visceral diseases (VL). By contrast, in *L. major *determining cutaneous infections (CL) we showed that A2 genes are present in a truncated form only. Furthermore, the A2 genomic sequences of *L. major *were considered subsequently to represent non-expressed pseudogenes [1]. Consequently, it was suggested that the structural and functional properties of A2 genes could play a role in the differential tropism of CL and VL leishmanias. On this basis, it was of importance to determine whether the observed structural/functional particularities of the *L. major *A2 genes were shared by other CL *Leishmania*, therefore representing a proper characteristic of CL A2 genes as opposed to those of VL isolates.

**Methods:**

In the present study we amplified by PCR and sequenced the A2 genes from genomic DNA and from clonal libraries of the four Old World CL species comparatively to a clonal population of *L. infantum *VL parasites. Using RT-PCR we also amplified and sequenced A2 mRNA transcripts from *L. major*.

**Results:**

A unique A2 sequence was identified in Old World cutaneous *Leishmania *by sequencing. The shared sequence was highly conserved among the various CL strains and species analysed, showing a single polymorphism C/G at position 58. The CL A2 gene was found to be functionally transcribed at both parasite stages.

**Conclusion:**

The present study shows that cutaneous strains of leishmania share a conserved functional A2 gene. As opposed to the multiple A2 genes described in VL isolates, the CL A2 gene is unique, lacking most of the nucleotide repeats that constitute the variable region at the 5'end of the VL A2 sequences. As the variable region of the VL A2 gene has been shown to correspond to a portion of the protein which is highly immunogenic, the present results support the hypothesis of a possible role of the A2 gene in the differential tropism of CL and VL leishmania parasites.

## Background

Leishmaniases are among the most important protozoan infections that affect humans in the world. The disease is widespread in 88 endemic countries with 350 million people at risk, 12 million people permanently affected, and an estimated annual incidence of 1.5–2 million cases [2]. This results in a global morbidity of 2,357 thousands DALYs (Disability Adjusted Life Years: number of healthy years of life lost due to premature death and disability) and a mortality rate of 59,000/year [3].

A surprisingly broad spectrum of clinical expressions is observed in humans ranging from asymptomatic to cutaneous (CL), diffuse cutaneous, mucocutaneous and visceral (VL) diseases, and an intermediary form known as post-Kala-Azar dermal leishmaniasis.

The various clinical issues of *Leishmania *infection depend on a complex host-parasite relationships where both the genetic or immunological status of the host [4-6] and the proper parasite biodiversity in terms of tropism and virulence [7,8] appear as determinant factors. A number of parasitic factors have been identified as susceptible to play a role in virulence/protection mechanisms in leishmaniases [9]. Among these, since its first identification in *Leishmania infantum *[10] several lines of evidence indicate that the A2 gene/protein family could be one of the most eligible candidate factor of virulence in VL infections: i) A2-proteins and mRNA transcripts are developementally expressed at the amastigote intracellular stage while undetectable in the promastigote [11], ii) Inhibition of A2-expression in *Leishmania donovani *using anti-sense RNA or by generation of partial knock-out mutants results in reduced virulence in vivo, iii) by contrast, increased parasite levels are observed in spleen of mice infected with A2-expressing transfected *L. major *[11,12] iv) A protective immunity can be achieved experimentally in mice by immunization with recombinant A2 protein or DNA vaccination showing that A2 from *L. donovani *is highly immunogenic and represents a potential antigen for protection in VL [13,14] and more recently in *L. amazomensis *infections [15].

A2 genes were detected by karyotype analysis in *L. donovani*, *L. infantum and L. chagasi *(Old World and New World VL) and in *L. mexicana and L. amazonensis *(New World DCL and MCL, respectively) but not in cutaneous species from the Old World (*L. tropica, L. aethiopica *and *L. major*) and the New World (*L. brasiliensis, L. guyanensis *and *L. panamensis*) [16]. Accordingly, A2-antibodies were found in sera from human and dogs naturally infected with *L. chagasi *(VL) [17] and in patients with VL in Sudan and India and CL due to *L. mexicana*, while they were not detected in *L. tropica *and *L. brasiliensis *infections (CL) [16].

While long considered absent in the *L. tropica *group, we identified by sequencing an A2 gene from crude PCR products of two strains of *L. major *(AF532102, AF532103) showing that the gene is present in *L. major *in a truncated form lacking most of the repeated motives that are present at the 3'end variable region of the VL A2 genes. Moreover, the *L. major *A2 gene was found subsequently to be non expressed and was considered to represent a pseudogene [1].

These observations raised the question of a possible role of the structure/functionality of A2 genes in the cutaneous or visceral tropism of *leishmania *parasites. As no data were available on the A2 gene of CL *Leishmania *except for *L. major*, our objective was to investigate this gene in Old World CL species. We amplified and sequenced A2-genes of additional strains of Old World CL species and in a clonal lineage of a *L. infantum *mediterranean strain isolated from a VL patient. Our results show that: i) The A2 sequence is extremely conserved both among strains and species of Old World CL *Leishmania*, ii) The CL A2 gene is a single copy gene of only 153 base pairs (bp) encoding for a protein of 51 amino acids, as opposed to A2 of VL species that are multicopy genes of varying length, ii) The CL A2 gene is functionally transcribed at the promastigote and amastigote stages.

## Methods

### Parasites

*Strains*. Six strains of one visceral and four cutaneous Old World *Leishmania *species were used for sequencing: *L. infantum *and *L. major, L. tropica, L. killicki *and *L. aethiopica*, respectively (Table I). Four of the five cutaneous strains were reference strains recommended by the W.H.O. [18]. In addition two *L. donovani *VL strains LEM3467 and LEM3566 were used for PCR analysis. All strains originated from the International *Leishmania *Cryobank and Identification Center, Montpellier, France.

**Table 1:** 

Tropism	Species	Strain	Zymodeme	Allele type	GeneBank accession number		
					
					gDNA^2^	mRNA	Protein^3^
Visceral (LV)	*L. infantum*	MHOM/FR/92/ LEM2385 Cl 1	MON-29	II	AY255807		AAP21103
				III	AY255808		AAP21104
				IV	AY255809		AAP21105
Cutaneous	*L. major*	IPAP/MA/86/ LEM898	MON-25	I.1	AF532102^2^	AY25581	AAM95954
		MHOM/SU/73/ 5 ASKH^1^	MON-4	I.2	AY185122		AAP21106
							AAO27297
(LC)	*L. aethiopica*	MHOM/ET/72/ L100^1^	MON-14	I.1	AY255804		AAP21100
	*L. killicki*	MHOM/TN/86/ LEM904- CL^1^	MON-8	I.1	AY255805		AAP21101
	*L. tropica*	MHOM/SU/74/ K27^1^	MON-60	I.1	AY255806		AAP21102

^1^WHO recommended reference strains. ^2 ^Direct genomic DNA sequencing.

^3^Predicted protein.

Strains originated from the International *Leishmania *Cryobank and Identification Center, Montpellier, France.

*Parasite clones*. Parasite clonal lineages were obtained from *L. infantum *MHOM/FR/92/LEM2385 and from *L. major *MHOM/SU/73/5 ASKH strains using a microplate technique as previously described [19].

*Culture and isolation*. Promastigotes were cultivated at 27°C in HOSMEM liquid medium [20] supplemented with hemin 10 μM (Sigma, Saint Quentin Fallavier, France) and 10% fetal calf serum (Gibco, Cergy-Pontoise, France). Parasites were inoculated into 25 ml culture flasks at day 0 (d0) at a final concentration of 10^5 ^ml^-1^. Amastigote organisms were isolated from foot-pad (*L. major*) or spleen (*L. infantum*) of Balb/c mice inoculated subcutaneously or intraveinously with 10^7 ^log-phase promastigotes, respectively. Parasites were washed twice in PBS and counted in Malassez chambers.

### DNA extraction

Washed parasites (100 μl PBS / ≈10^9 ^parasites) were lysed by thermal shock in Eppendorf tubes, 1 mn in boiling water – 1 mn in melting ice, three times. DNA extraction was performed using classical phenol/chloroform/isoamylic alcool protocol and precipitation was made using NaCl/ethanol procedure. The DNA was dissolved in 40 μl of sterile water.

### PCR and sequencing

Amplification of the parasite DNA matrix (50 ng) was made using L2/R3 primers (5'-TTGGCAATGCGAGCGTCACAGTC / 5'- CAACGCGTACGATAATGCCACA). The L2/R3 primers correspond to the 5' end position 16301 and 16603 of the inverse-complementary strand of the AC010851 sequence, respectively. The PCR was performed in a reaction mixture of 50 μl containing either 1 or 3 mM MgCl_2_, 200 μM each dNTP, 25 pmol of each primer (Proligo, Paris, France), 1 U Taq polymerase (Eurogentec, Seraing, Belgium). L2/R3-PCR conditions consisted to denaturation for 3 mn at 94°C, followed by 35 amplification cycles at 94°C for 1 mn, 1 mn at 58°C, 1 mn at 72°C, then one cycle at 72°C for 5 mn. Amplification of cDNA from bacterial culture medium (0.5 μl) was made using M13 forward-20 / M13 reverse (Qiagen PCR cloning kit, Qiagen, Courtaboeuf, France) with 1 mM MgCl_2_. M13 forward-20 / M13 reverse -PCR conditions consisted in a hot-start denaturation for 10 mn 95°C, followed by addition of 1 U Taq polymerase, 30 amplification cycles at 94°C for 30 sec, 30 sec at 48°C, 1 mn at 72°C, then one cycle at 72°C for 5 mn. Five microliters of PCR product was electrophoresed in 2% agarose gel in the presence of ethidium bromide, and visualized under UV light. A 50-bp ladder (Sigma) was used as MW marker.

For sequencing, the two strands of PCR-amplified DNA were purified with QIAquick PCR Purification Kit (Qiagen) and sequenced with the corresponding PCR primer set using the BigDye Terminator Sequencing Kit V3.1 (Applied Biosystems, Courtaboeuf, France) on an automated sequencer 3100 Genetic analyser (Applied Biosystems).

### RNA extraction and reverse transcription-PCR (RT-PCR)

*L. major *IPAP/MA/86/LEM898 total RNA was extracted from promastigote cultures or infected organs (≈10^6^-10^7 ^parasites) using Rneasy Plant Mini Kit (Qiagen). To eliminate any remaining DNA the RNA extract mixture (5 μl) was additionnaly treated by Dnase Rnase-free (Eurogentec) for 30 min at 37°C in a final volume of 30 μl. As a negative control, an aliquote of the sample (5 μl) was subsequently digested with Rnase A (Qiagen) 700 mg 1 h at 25°C in a final volume of 16 μl. RT was performed in a total volume of 20 μl by 50-min incubation at 42°C followed by 15 min at 70°C to inactivate the reverse transcriptase. The reaction mixture contained a sixth of the initial volume of the RNA extraction products, and the following final reagent concentrations: 1X hexanucleotide mix (Roche, Meylan France), 500 μM dNTP mix, 40U Rnase inhibitor, 1X first strand buffer, 100 mM dithiothreitol, and 200 U Super Script II (Invitrogen, Cergy Pontoise, France). Two microliters of RT products were PCR-amplified with L2/R3 primer set.

### DNA libraries

L2/R3-PCR products from genomic DNA matrix were synthetized as described above except for the MgCl_2 _concentration (3 mM). PCR products were purified on QIAquick column (Qiagen). Poly-A treatment, insertion of the PCR products into pDrive vector, transformation of *E. coli *EZ competent cells (Qiagen) and cloning were performed using a PCR Cloning Kit (Qiagen) as described by the supplier. A2-containing genomic clones were screened by digestion of M13-PCR products with Sau3AI endonuclease (BioLabs, Saint Quentin en Yvelines, France) which cuts off A2-gene nucleotidic sequences at position 33–34 (reference : *L. major *MHOM/IR/-/173; AF532103).

### GeneBank accession numbers

Accession numbers for genomic DNA, mRNA and putative protein sequences are given in Table I.

## Results

### PCR

**Figure 1:**
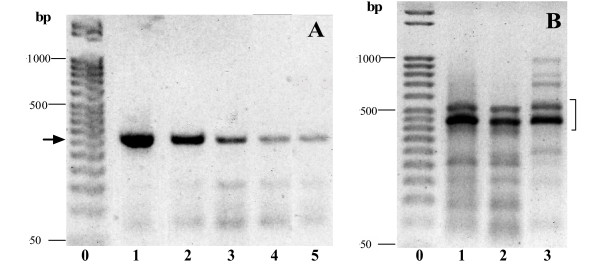
**PCR electrophoresis patterns. **Electrophoretic patterns of PCR products obtained from crude parasite genomic DNAs using 3 mM MgCl_2_. **A**. CL isolates: 1, *L. major *LEM898; 2, *L. major *LEM134; 3, *L. aethiopica *LEM144; 4, *L. tropica *LEM419; 5, *L. killicki *LEM904; Light arrow: A2 gene. **B**. VL isolates: 1, *L. infantum *LEM2385-cl1; 2, *L. donovani *LEM3467-cl3 3; *L. donovani *LEM3566; square bracket: A2-gene area.

L2/R3 PCR products obtained from parasite crude genomic DNA resolved in different electrophoretic patterns, according to the species. For all CL isolates (Fig. 1-A) one single band of about 260 bp was evidenced when PCR was performed using stringent or not stringent conditions (1 or 3 mM Mgcl_2_), thus these products were available for direct sequencing. By contrast PCR amplification products could be obtained only under non stringent conditions (3 mM MgCl_2_) for VL species, resolving in a complex electrophoresis pattern (Fig. 1-B). These products were shown by direct sequencing to be a mixture of A2 sequences and of non specific products resulting probably from a certain degree of mispriming due to non stringent conditions. As a consequence direct sequencing of L2R3 PCR products from crude genomic DNA of VL species could not be performed, thus A2 sequences were obtained from clone libraries of *L. infantum *MHOM/FR/92/LEM2385-clone 1.

The PCR performed with M13 primers on clone libraries corroborated the above results. All genomic-DNA clones originating from *L. major, L. tropica, L. aethiopica and L. killicki *resolved in a unique band of 300 bp. By contrast, three different bands of 370, 410 and 460 bp were identified from the *L. infantum *clone library (data not shown). Only 1/4 of the library clones (20 clones were sequenced) corresponded to cloned A2-gene. The other clones corresponded to unknown or microsatellite structures (3 clones) in BLAST analysis study.

### Sequencing (Fig. 2)

**Figure 2:**
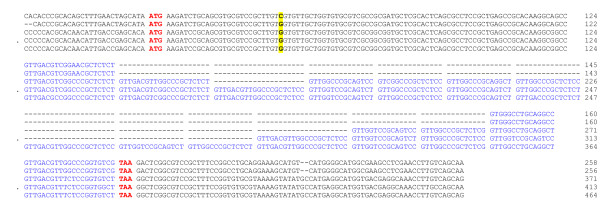
**A2 nucleotide sequences. ***Leishmania major *IPAP/MA/86/LEM 898 and MHOM/SU/73/5 ASKH LEM134 (allele types I.1 and I.2) and *Leishmania infantum *MHOM/FR/92/LEM2385 clone 1 (allele types II to IV). Internal nucleotide repeats are shown in blue. The polymorphism C/G at position 58 is highlighted in yellow.

#### Old-World CL strains

A single sequence of 258 nucleotides was evidenced by directly sequencing the crude L2/R3-PCR products of the genomic-DNA of the three *L. major *strains. The three sequences were found identical with the exception of a single polymorphism C/G at position 58 for the *L. major *134 and 173 as compared with *L. major *898. Additional nucleotide sequences from 32 genomic clones of the above mentioned *L. major *strains were all identical to the corresponding crude sequences. Moreover, the sequences obtained from crude L2/R3-PCR products and from 15 genomic clones of *L. aethiopica*, *L. tropica *and *L. killicki *were totally identical to the *L. major*-898 sequence. These sequences were referred to as A2-gene allele typeI.1 and I.2, respectively.

#### L. infantum VL strain

Three A2-gene sequences of 371, 413 and 464 nucleotides were isolated from the genomic library of *L. infantum *MHOM/FR/92/LEM2385 clone 1 (*L*. *infantum*-2385.1). These sequences were referred to as A2 type II-, III- and IV-alleles.

Comparative analysis of A2-gene alleles from CL (type I) and VL (Type II, III and IV) strains showed that the genes are composed of a common nucleotide sequence at the 5' end followed by a region of varying length inserted from the position 97 of the ORF to the 3' end which is a stretch of a number of more or less conserved repeated nucleotide patterns.

### mRNA expression

**Figure 3:**
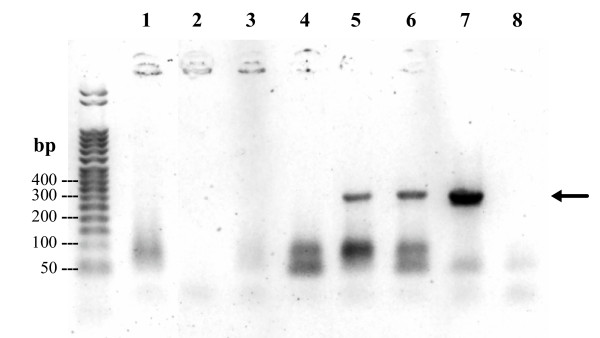
**RT-PCR on IPAP/MA/86/LEM898 strain Dnase-treated mRNA extracts. **Additional Rnase-digestion (1–3). Uninfected spleen (1,4); Cultured promastigotes (2,5); Foot-pad (3,6). Controls: Genomic DNA (7); H_2_O (8). Arrow : A2 mRNA transcripts.

Results of RT-PCR on Dnase-treated *L. major *IPAP/MA/86/LEM898 mRNA extracts are presented in Fig. 3. A single band was evidenced in RT-PCR products from both promastigotes and foot pads of infected mice. A faint band was also observed in popliteal lymph node extracts (data not shown). The sequencing of the RT-PCR cDNA product resulted in a 258-nucleotide sequence (AY255810) identical to the sequence obtained from the crude genomic DNA, and corresponding to a putative A2 protein of 51 AA.

## Discussion

*Leishmania *A2-genes were first identified in two strains of the *L. donovani *complex, *L. infantum *LV9 and *L. donovani *1S2D determining visceral infections [10,21]. In these VL strains A2 genes were shown to be organized in several clusters each comprising multiple A2 genes of varying length that are tandemly associated with related sequences (A2rel) [10,11,22]. However, these results were obtained from leishmania strains isolated from naturally infected hosts which are known to be most likely composed of multiple parasite populations [23,24]. Therefore, the existence of multiple A2 genes remained to be confirmed using a genetically pure parasite clonal lineage. As in the present study three different A2-alleles type II, III and IV were sequenced from the *L. infantum *MHOM/FR/92/LEM2385 Clone-1 genomic library, our results provide additional evidence that A2 of VL species is a multiple gene family. Alleles type I, II and III differ only in the number and arangement of the repeated motives at the 3'end variable region of the gene as previously described in VL strains [10]. However, in the present study we identified A2 sequences showing a limited number of repeats and consequently a length of only 371 to 464 bp contrasting with the previously published A2 genes of about 700–800 bp. The inability to evidence A2 sequences > 1 kb in the present study is most probably due to the limits of the PCR technique performed on crude genomic parasite DNA or to the absence of long A2 sequences in this strain. Thus these results are not contradictory to the previously published data but bring additional information on the variability of the *L. infantum *A2 genes.

We previously identified A2 sequences in two strains of *L. major *IPAP/MA/86/LEM898 and MHOM/IR/00/173 (AF532102 and AF532103, respectively). These sequences were 95% (88 nt/93) identical to the S69693 stage-specific S antigen homolog (A2) of *L. infantum *VL [10] at the 5'-end of the ORF (nucleotides 74 to 167). By contrast, a major deletion of the 3'end variable region of repeated nucleotide motives was observed in *L. major *A2 genes.

In the present study, the amplification of Old World *Leishmania *genomic DNA with L2/R3 A2-gene primer set resolved in a single amplification product on gel electrophoresis, as opposed to the complex pattern observed with *L. infantum*. Accordingly, a single sequence of 258 nucleotides was obtained by direct sequencing crude DNA products from all CL *Leishmania*, whatever the strain or species. A common sequence was also isolated from 32 genomic clones of *L. major *LEM898 and 15 clones of *L. tropica, L. aethiopica and L. killicki*. The A2 sequence shared by all strains and species of Old World *leishmania *presented a single polymorphism C/G at position 58 of the ORF. These results show for the first time that the A2 gene of Old World cutaneous *Leishmania *is unique and highly conserved, contrasting strongly with the multiple A2 sequences of varying length observed in VL isolates.

RT-PCR on mRNA extracts from strain IPAP/MA/86/LEM898 followed by sequencing evidenced that the CL A2 gene is functional. It is noteworthy that this finding does not presume of the expression of the protein at the post-transcriptional level, however it contrasts with the previous suggestion based on the failure to demonstrate A2 gene transcripts in *L. major *that the A2-gene of *L. major *is a non-expressed pseudogene [1]. RT-PCR amplification of both promastigote and amastigote mRNAs resulted in a similar signal on gel electrophoresis in the present study, showing that *L. major *A2-gene is transcribed at both amastigote and promastigote stages. However, these results do not signify that *L. major *A2 are not developmentally expressed since RT-PCR is not quantitative. Actually, *L. infantum *promastigotes were reported to express very low levels of A2 [10,22].

## Conclusion

The present study evidence that Old World cutaneous species of *Leishmania *share a common, highly conserved and functional A2 gene. In CL *Leishmania *the A2 gene is a single gene in which the 3'end variable region is almost entirely deleted, contrasting with VL A2 genes that are a family of multiple genes where the 3'end portion is a variable stretch of nucleotide repeats. Further investigations are needed for exploring the potential role of theses structural differences between CL and VL A2 genes in governing both the proper parasite virulence/tropism and host susceptibility/protection response conditioning the multiple clinical issues observed in human *Leishmania *infections.

## Competing interests

The author(s) declare that they have no competing interests.

## Authors' contributions

YJFG conceived and conducted the study and drafted the manuscript. PM carried out the work at the technical level. FP and JPD supplied and characterized the parasite strains. FD is the Chief Manager of the Laboratory and revised the manuscript and FL directed the biomolecular and bioinformatic analyses and participated to the writing of the manuscript.

## Pre-publication history

The pre-publication history for this paper can be accessed here:


